# A randomized controlled trial of Internet-delivered guided and unguided cognitive behaviour therapy for treating depression and anxiety in UK university students: study protocol for the Nurture-U Internet CBT trial

**DOI:** 10.1186/s13063-025-09023-1

**Published:** 2025-09-26

**Authors:** E.R Watkins, D Phillips, H Choueiri, A Ford, H Cook, G Taylor, R.C Kessler

**Affiliations:** 1 Mood Disorders Centre, School of Psychology, Sir Henry Wellcome Building for Mood Disorders Research, University of Exeter, Exeter, EX4 4LN UK; 2 Clinical Trials Unit, Medical School, University of Exeter, University of Exeter, Exeter, UK; 3 Department of Health Care Policy, Harvard Medical School, Boston, MA USA

**Keywords:** Depression, Anxiety, University students, ICBT, Randomized controlled trial, Precision treatment algorithm

## Abstract

**Background:**

Tackling poor mental health in university students is a priority in higher education. Although major depressive disorder and generalized anxiety disorder are highly prevalent among university students and predict impaired university and later life outcomes, most students do not receive evidence-based treatment. Internet-delivered cognitive-behavioural therapy (iCBT) is increasingly offered to UK university students in guided or unguided formats. Our aim is to compare the effects of guided versus unguided scalable iCBT for university students with elevated symptoms of depression and/or anxiety and to investigate the feasibility of creating individual treatment rules (ITR) to predict for whom which variant of iCBT is more effective as well as for whom neither is effective.

**Methods:**

An online single-blind, two-arm parallel-group randomized controlled trial to examine symptoms of depression and anxiety across 3 months in university students aged over 16 who screen into the study with self-reported high levels of anxiety (GAD-7 > 9) and/or depression (PHQ-9 > 9). Eligible participants will be randomized to guided transdiagnostic iCBT supported by a psychological wellbeing practitioner or to the same transdiagnostic iCBT content as unguided self-help. In total, 720 participants with no current bipolar disorder or psychosis will be recruited from UK universities. Assessments will take place at baseline (pre-randomization) and 3 months post-randomization. Primary endpoints and outcomes are self-reported depression and anxiety symptoms at 3-month follow-up, adjusting for baseline scores. Well-being, health-related quality of life, functioning and academic outcomes are secondary outcomes. Compliance, adverse events, and potentially mediating variables will be monitored. We will use machine learning to estimate heterogeneity of treatment effects and develop an ITR to optimize the allocation of students to either unguided or guided iCBT.

**Discussion:**

The trial aims to provide a better understanding of the relative benefits of guided and unguided iCBT for anxiety and depression in university students with considerable implications for treatment coverage and service planning and delivery. We will provide innovative information to optimize treatment assignment, guide university mental health treatment planning and support evidence-based and scalable interventions for the most common mental health problems in university students.

**Trial registration:**

ISRCTN: 56784470, https://www.isrctn.com/ISRCTN56784470. Registered on 27 October 2022.

**Supplementary Information:**

The online version contains supplementary material available at 10.1186/s13063-025-09023-1.

## Background

Improving student mental health at university has increasingly been identified as a priority amongst both researchers [1–5] and key stakeholders [6–9]. Critically, there is increasing evidence of the high prevalence of anxiety and depression amongst university students globally [5, 10–12], with a series of international surveys finding that 25–40% of students report elevated anxiety and/or depression, often at a clinical level [4, 10–12].

In parallel, university mental health services report a dramatic increase in the number of students seeking help for mental health concerns and a growing disparity between student demand and current resources [5–9]. Since most United Kingdom (UK) students with mental health problems access services via their university rather than the National Health Service (NHS), there is now a major treatment gap. The organization, provision and means for students to access mental health services vary across institutions. None of the different service models nor most student mental health initiatives have been systematically evaluated and are often applied without a direct evidence base.

The recent UK University Mental Health Charter [8], a product of research synthesis, stakeholder consultation, and student co-creation, recommended a systemic whole-university approach to mental health, arguing that institutional environment, organizational culture, course content and delivery are all important for mental health, along with the integration of promotion and prevention and the provision of specialist support (see also 5,7). In parallel, recent reports [5] and the Universities UK StepChange framework [9] recommended stepped care tailored to the university environment to address the capacity and complexity of university mental health support needs. Stepped care typically involves access to and movement between different levels of intensity of intervention based on patient need and presentation [13].

One potential component within a stepped care model is the stepping up from through different intensities of therapy, with completely unguided self-help interventions such as unguided digital cognitive-behavioural therapy (CBT) offered for individuals with the mildest symptoms, the same digital CBT supported and guided by trained professionals, i.e. guided self-help, offered to individuals with mild-to-moderate symptoms, and with intensive face-to-face counselling or CBT offered to individuals with moderate-to-severe depression. Low-intensity (LI) self-help CBT, with or without support from a mental health professional, delivered via books or digitally is proven effective for treating common mental health difficulties [14–18], improves access and reduces delivery costs (e.g. the NHS Talking Therapies programme). Further, digital self-help is highly scalable and usable anywhere, anytime, potentially addressing treatment barriers and challenges around treatment access, capacity, convenience, and availability.

Digital CBT for mild-to-moderate anxiety and depression is found to be efficacious in students, albeit with small average effect sizes [19, 20]. However, most trials in students have only involved small samples (less than 100 participants per arm) and only a handful have selected participants with elevated anxiety and depression [21, 22]. Students generally view online interventions positively [23]. Despite this, self-help CBT has been less frequently evaluated within university student mental health support services, and when used, does not always adhere to established LI-CBT principles. There is an increasing use of digital CBT in UK universities as part of the portfolio of wellbeing services, often delivered in an unguided format, despite evidence that guided versions of CBT typically outperform unguided CBT interventions [17, 24, 25]. However, because co-morbidity of anxiety and depression is highly prevalent amongst university students, digital CBT interventions need to be sufficiently transdiagnostic to tackle both anxiety and depression, although most trials have focused separately on interventions for either anxiety or depression. To address these gaps, we plan the first large-scale trial in the UK of iCBT for students that will select students with elevated anxiety and/or depression scores, utilize transdiagnostic CBT, and compare guided versus unguided iCBT, relevant to improving stepped care approaches.

A key uncertainty within digital self-help is the heterogeneity of treatment effects; that is, variations in individual treatment response to different treatments [19, 24]. Understanding which digital self-help interventions are most acceptable and work best for which students could help to tailor self-help interventions, plan the care pathway, and improve outcomes. A critical question is how much and what kind of support from a practitioner is needed: unguided self-help has greater reach, sustainability and scalability as it is not limited by therapist capacity, opening up the potential for massive cost-effective open online interventions [26] but may have less engagement and efficacy than guided interventions [17, 20, 24, 25, 27, 28], although there is some heterogeneity [19, 24], with a subset of individuals benefiting as much from unguided self-help as guided self-help [25, 29]. Identifying such a subset would be of enormous value in increasing treatment scalability.

An emerging approach to develop precision predictive models is to use machine learning. A recent study used this approach to develop individualized treatment rules (ITR) for university students with anxiety or depression in Mexico and Colombia who had participated in a pragmatic trial comparing guided iCBT versus unguided iCBT versus treatment-as-usual [27, 30]. Machine learning enables the estimation of a prediction model for each treatment arm, which can then be used to determine counterfactual predicted outcomes for each student for guided i-CBT relative to unguided i-CBT and then to compare the difference between each predicted outcome to decide which treatment is best for each student. Such information could be very useful for deciding what type of treatment to offer different types of students and for planning of student treatment services.

Benjet et al. (2023) [30] used a machine learning precision treatment modelling method [31] to develop an ITR. We propose adopting a similar approach,thereby, to our knowledge, making the current study the first application of this approach to a European university student sample. This provides an opportunity to examine if similar patterns of heterogeneity of treatment effects and similar ITRs emerge in a UK higher setting compared to those found in Latin American higher education [27, 30]. Following the precedent of Benjet et al. (2023) [30], we will assess an extensive set of potential predictors of individual differences in the effects of CBT, that is, potential prescriptive predictors [32], moving beyond prior research that only examined a small battery of predictors [33]. As such, we aim to test whether it is possible to generate a useful ITR for guided versus unguided i-CBT in the UK university context.

### Objective

The primary objective is to examine the aggregate efficacy of guided transdiagnostic i-CBT relative to unguided i-CBT to reduce symptoms of depression and anxiety over a 3-month period in university students who report elevated depression and/or anxiety, as operationalized by caseness on well-established self-report measures. As noted above, we know from much previous research that the aggregate efficacy of guided i-CBT is likely to be significantly higher than that of unguided i-CBT. A secondary objective is to use machine learning methods to predict heterogeneity of treatment effects of unguided iCBT versus guided iCBT and develop ITRs to predict which students will respond as well as, or possibly even better than, to self-guided as to guided i-CBT.

We hypothesize that consistent with prior findings [17, 20, 24, 25, 27, 28], for university students with elevated depression and/or anxiety, the guided i-CBT will outperform unguided i-CBT in the aggregate in reducing symptoms of depression and anxiety across 3 months. We further hypothesize that a small subset of users will benefit at least as much from unguided as from guided i-CBT and that we will be able to identify this subset of students prior to randomization using machine learning methods [30]. Importantly, we also anticipate that some proportion of students will fail to improve meaningfully with either guided or self-guided i-CBT and that we will be able to identify this segment of the population with good accuracy using the same machine learning measures as those used to develop the ITR. If so, this information could be used to refer such students to more intensive care rather than requiring them first to fail as a course of i-CBT.

## Methods

The study will be conducted and reported according to Consolidated Standards of Reporting Trials (CONSORT) [34, 35] and extensions for non-pharmacologic treatment interventions and multi-arm parallel-group randomized trials and CONSORT-EHEALTH for improving and standardizing evaluation reports of Web-based and mobile health interventions [36] (see accompanying SPIRIT checklist).

### Study design

The trial design is a superiority parallel 2-arm individual-level single-blind (with researchers following up participants and statisticians conducting analysis blind to treatment allocation) randomized controlled trial (RCT). All participants will be randomized with equal allocation to receive either guided or unguided i-CBT.

Potential participants for the trial will provide initial consent to complete screening measures to determine if they are eligible to participate in the trial, i.e. showing elevated anxiety and/or depression or current diagnosis of major depression. Any potential participants who are found not to be eligible will automatically be signposted to other sources of support. Once trial eligibility has been determined and consent to participate in the trial has been obtained, participants will be individually selected at random (in a 1:1 ratio) to be offered guided i-CBT or unguided i-CBT.

### Study settings

We seek to recruit 720 participants from within UK universities. Because all aspects of the trial are conducted remotely, there is a single active central study site delivering the study, namely the University of Exeter, which provides the coordinated central hub through which participants will be screened, randomized, data collected, treatment offered, and follow-up assessments delivered.

### Recruitment

There are two approaches to recruitment. First, there is direct advertising of the study to students within the partner universities in the grant (Exeter, Newcastle, Oxford, Cardiff, Southampton, and King’s College London) including on-campus presentations, posters, stands, and presentations in lectures within the partner universities, and email to mailing lists of students and use of newsletters, other circulars, and noticeboards within the partner universities. Second, there is a social media campaign advertising direct to university students across the UK and open to students attending other universities. This social media campaign will be designed and prepared to be carried out on different social networks (e.g. Facebook, Instagram, TikTok), including advertisements in social media direct to university students across the UK. This approach is selected to increase the range of recruitment and increase the generalizability of the students recruited into the trial.

### Eligibility criteria

Eligible participants will be as follows: (1) over 16 years of age; (2) studying at a UK university; (3) able to provide informed consent; (4) having regular access to a smart phone (android or iOS), tablet, PC or laptop, necessary to use the intervention; (5) available for the full duration of the trial (3 months); (6) able to complete consent and online questionnaires based on a basic literacy in English; (7) scoring above previously established clinical caseness threshold cut-offs on a standardized self-report measure of either anxiety (Generalized Anxiety Disorder-7, GAD-7, > 9) [37] and/or depression (Patient Health Questionnaire-9, PHQ-9, > 9) [38] or meeting current diagnostic criteria for major depression (according to psychiatric DSM-V criteria), determined in structured self-report electronic screening using the LIDAS instrument [39]. Exclusion criteria will include: active suicidality (operationalized by endorsing thoughts of death or hurting oneself more than half the days over the last 2 weeks on PHQ-9 question 9 and answering yes to at least one subsequent automatic contingent question about either suicidal intentions or plans); any self-reported history of severe mental health problems such as bipolar disorder, psychosis, or drug/alcohol dependence; and currently receiving psychological therapy, counselling or any type of psychiatric medication (operationalized as being received at the point of seeking entry to the study).

### Screening and consent procedure

Potential participants who are interested in the study will be directed to our study website, (www.nurtureuniversity.co.uk) which provides further information. Interested participants can proceed directly to the Electronic Data Capture (EDC) system and undertake a brief pre-screener to confirm age and enrollment as a university student. If appropriate, the website visitor can watch a video version of the screening Participant Information Sheet (PIS) and/or read an electronic version of the PIS and an initial consent screen to provide contact details (email including a UK university email address ending in “.ac.uk” and separate personal email address; mobile phone number), and to provide informed consent (screening consent form) to complete the screening questionnaires. After completing pre-screening, potential participants will be automatically emailed a copy of the screening PIS, privacy policy and completed screening consent form. Once this initial consent is provided, the participant will complete the screening assessment consisting of the LIDAS, PHQ-9, GAD-7, Penn State Worry Questionnaire (PSWQ-S) [40], Ruminative Response Scale-Brooding subscale (RRS-B) [41] and if meeting eligibility criteria will be assigned to the trial. The screening process will in parallel assess participants for eligibility for a separate trial of an app targeting repetitive negative thought for students with elevated worry and rumination but who are not currently depressed, hence the inclusion of RRS-B and PSWQ-S measures: students reporting scores below required cut-offs for depression symptoms and diagnosis but with elevated worry and rumination will be filtered to this other trial [42].

Individuals meeting eligibility criteria following the screening assessment will then be asked to consent to take part in the trial after viewing a video version of the trial PIS and/or reading an electronic version of the trial PIS and completing an electronic trial consent form (trial consent form). Participants who provide consent are automatically emailed a copy of the trial PIS, privacy policy and completed trial consent form.

Individuals who are not suitable at pre-screen or screening (e.g. outside of age range, not have elevated anxiety or depression) will automatically be directed to a webpage explaining why they are not suitable for the trial. Those reporting mental health difficulties will be automatically guided to webpages providing information, guidance including consulting with their general practitioner (or equivalent), and weblinks and telephone numbers for help and support, including contact details for the trial team.

### Baseline and follow-up assessments

**Fig. 1:**
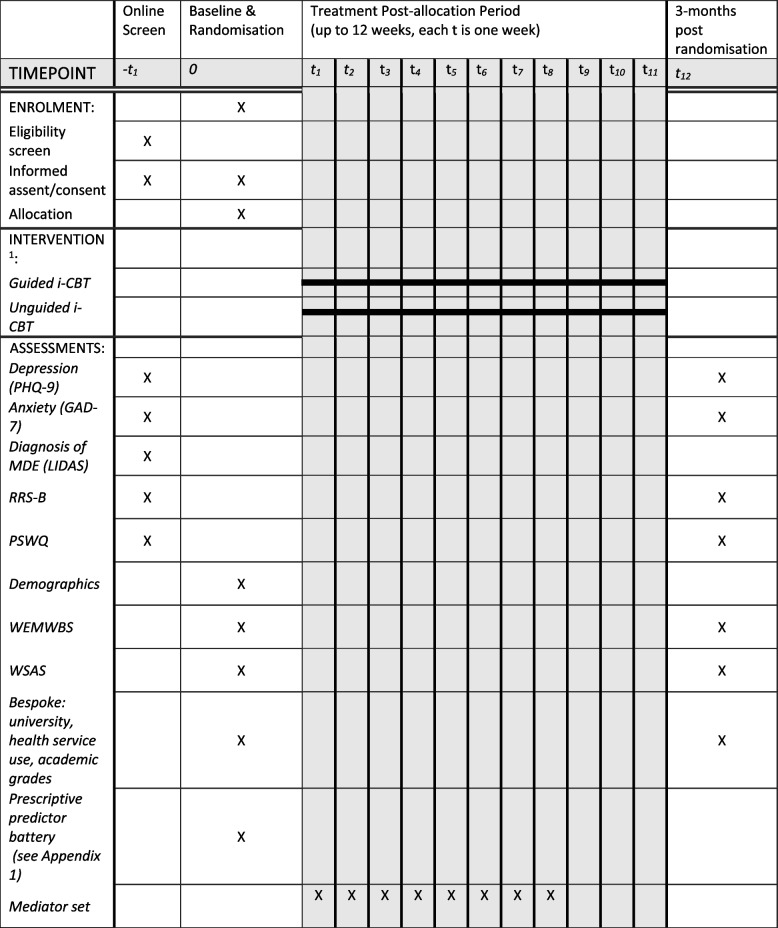
Schedule of enrolment, interventions, and assessments (SPIRIT figure)

The baseline assessment will take place after electronic informed consent to participate in the trial is provided and consists of web-based self-report measures to assess current and lifetime history of depression, current wellbeing, symptoms of anxiety and depression, social and work functioning, use of services and treatment received, academic grades, resilience, and stressful events (see outcome measures and Fig. 1—SPIRIT figure, Table 1). The Lifetime Depression Assessment Self-report questionnaire (LIDAS) [39] will be used to assess lifetime major depression (MDD) diagnosis according to DSM criteria and is largely based on the widely used Composite International Diagnostic Interview (CIDI). The LIDAS has been proven to be effective for determining history of depression through self-report in an online digital format, matching the needs for the current study [39]. The instrument consists of a conditional sequence of pre-programmed questions assessing all the diagnostic criteria for depression, with logic cut-outs so that subsequent questions are determined by prior questions, keeping the assessment brief.

**Table 1:** Measurements and endpoints

**Web Assessment**		**Baseline**	**Follow-up (3 months)**
Pre-screening	date of birth, self-reported mental health	YES	NO
Informed consent			
Socio-demographics	Age, sex, employment status, ethnicity, historic mental health problems	YES	NO
Depression	PHQ-9 questionnaire; primary outcome	YES	YES
Anxiety	GAD-7 questionnaire; primary outcome	YES	YES
LIDAS	Incidence of current and past major depressive episode	YES	NO
Rumination	RRS-Brooding questionnaire; secondary outcome	YES	YES
Worry	PSWQ questionnaire; secondary outcome	YES	YES
Wellbeing	WEMWBS questionnaire; secondary outcome	YES	YES
Resilience	BRS questionnaire; secondary outcome	YES	YES
Perceived stress	PSS-4 questionnaire; secondary outcome	YES	YES
Stressful events	ASQ questionnaire; secondary outcome	YES	YES
Use of mental health services and resources		NO	YES
Academic outcomes		YES	YES

The baseline assessment will also include an extensive battery of potential prescriptive predictors to support the development of the ITR. The selection of instruments will be adapted for a UK context from those used by Benjet et al. (2023), which were originally chosen following a systematic literature review of prescriptive predictors of heterogeneity of anxiety and depression treatment response with respect to CBT to identify measures that had predicted treatment outcomes in prior studies [27, 30, 43]. Such prescriptive predictors include demographics, mental health history and treatment, co-morbid symptoms, stress, personality and coping styles. Constructs to be assessed will include: Past history of depression,functional impairment; depression and anxiety symptoms; self-reported cognitive functioning, anger/irritability; mental health history, sleep problems; screening for alcohol and substance use, generalized anxiety, panic, posttraumatic stress disorder, bipolar disorder; suicidal ideation, suicide attempt; lifetime mental health treatment (age of onset, duration, type, hospitalizations); views on iCBT including perceived efficacy, anticipated adherence and orientations towards digital technology and interventions, goals for intervention, user treatment preferences; family history of mental/emotional problems; adverse childhood experiences; current stress severity, recent stressful life events, stress reactivity; agreeableness; alexithymia; conscientiousness; emotionality; extraversion; hopelessness; neuroticism; openness; attachment style; self-esteem; problem-solving ability; resilience; social support; loneliness. For fuller details on relevant measures, see Appendix 1.

Once consented participants have completed the baseline assessment, they will be randomized automatically by an independent computer system. Randomized participants will be informed of their randomization allocation and signed up to use the relevant variant of the Internet platform via clinically trained members of the research team. The participant’s university email address will be used for Internet platform set-up.

**Fig. 2:**
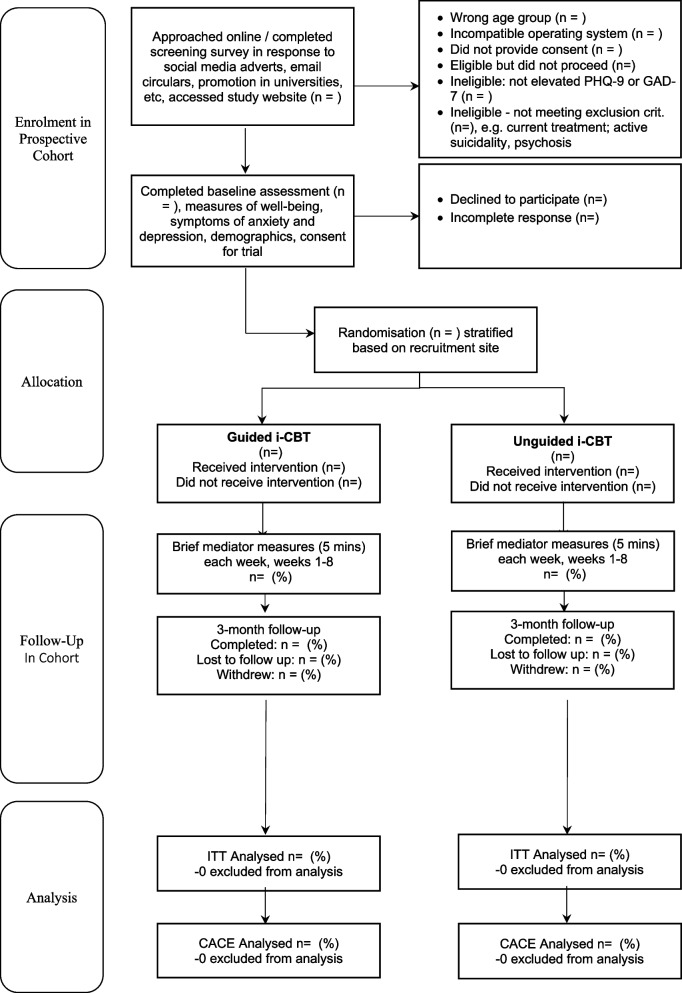
CONSORT flow diagram

All participants will be followed up electronically at 3 months post-randomization. At the follow-up point, participants will be automatically sent emails and texts with links to enter their data into the EDC. Each assessment point will involve an automated weekly follow-up by email and then text and telephone follow-ups to participants who haven’t yet completed the EDC assessment at 3 months. Figure 1 and Table 1 give an overview of all measurements. Figure 2 gives an overview of trial flow. Project researchers will be blind to treatment allocation but will be available to participants to answer queries about the trial or the assessments. Participants will receive honorariums for the completion of each of the brief mediator assessments occurring each week for 8 weeks post-randomization, and at 3-month follow-up assessment.

### Randomization, intervention delivery, and masking

Participants will be randomized (in a 1:1 ratio) to the two intervention arms. Randomization will be conducted automatically by means of a custom-built secure web service created and managed by the Exeter Clinical Trials Unit (ExeCTU), which interfaces with the trial database and which will be independent of the trial researchers. To promote balance across key participant characteristics across intervention arms, randomization will be stratified according to recruitment site (each of the six funded partner universities plus “other” university category for students directly recruited via social media advertising). Blocking will be used with a minimum block size of 4 and a maximum block size of 6, occurring at random to maintain concealment.

All the online recruitment and randomization will be automated and independent of trial researchers. The Exeter CTU EDC system will contact an unblinded team member (administrator/therapist) indicating when an individual [by study ID] has been randomized to the active intervention. This team member will then access the relevant details in the ExeCTU database and manually set up the participant in the i-CBT platform using administrator rights via the treatment platform dashboard. An email will also go to the participant from the clinically trained members of the team providing support to the i-CBT, indicating the condition to which they are randomized and informing them what to expect. These team members will also monitor if the participant has accessed the i-CBT platform and check if any difficulties arise to encourage sign-up.

All assessments will be routinely collected online using the EDC following automated reminders, without the involvement of researchers. Site researchers will be blind to treatment allocation and analyses will be undertaken by a statistician blinded to group allocation. Site researchers will prompt all participants to complete follow-ups by email, text, WhatsApp and telephone call if they do not respond to the automated email reminders, including as a final step seeking to ask participants to complete the primary outcomes only over the telephone with the researcher. Any unblinding in contact with a site researcher would be logged as a protocol deviation and only a researcher that remained blind will be able to prompt future follow-up from that participant.

## Interventions

### Guided and unguided i-CBT

The treatment will be an established Internet platform that provides a standard transdiagnostic cognitive-behavioural approach to treating anxiety and depression, including proven elements such as activity scheduling, exposure to anxious situations, psychoeducation, thought challenging, behavioural experiments, relaxation and problem-solving. We chose the i-CBT platform developed by SilverCloud Health because (a) it has been widely used commercially [44] and in the NHS in England, (b) has evidence of efficacy within clinical trials [45, 46] including within student populations (21, 27), (c) is currently used by at least 20 UK universities and (d) was the intervention used by Benjet et al. (2023) [27, 30], thereby enabling direct comparison between the current research and the similarly designed trial conducted in Colombia and Mexico. The specific interventions to be used will be the SilverCloud Space from Anxiety, Space from Depression, and Space from Depression and Anxiety packages. Because this is a pre-programmed digital intervention, it has fixed intervention content within its modules and, as such, the intervention is not modifiable during the study.

The i-CBT will be delivered online and can be accessed on smartphone, tablet, PC, or laptop to maximize means to access self-help, to benefit from increased engagement by allowing users to choose their preference of which to use. The intervention includes written text, pictures, videos, audio-exercises, questionnaires, quizzes, and interactive tools. It is organized into 8 core modules and 12 supplemental modules (see Table 2 for further details) that include evidence-based strategies for cognitive restructuring, behavioural activation, relaxation training, and graded exposure.

**Table 2:** Detailed description of internet CBT intervention

Module name and number	Content	Exercises and tools	Treatment
1. Getting started	• Welcome to SilverCloud (introductory video) • How can CBT help you? Text psychoeduction • Making Changes (video) • The Key to CBT (video) • Personal stories (audio) • Introduce personal stories • Summing up	• Mood Monitor (mood record to complete) • My CBT Cycles (tool to examine relationship between thought, feelings, behaviour) • Staying in the Present (audio-exercise) • Steps towards your goals (goal-setting)	Common to Space from Depression, Space from Anxiety, Space from Depression and Anxiety
2. Understanding depression and anxiety	• Space from Depression and Anxiety (video) • Myths and facts quiz • Learning more: Depression and Anxiety psychoeducation • The Cycle of depression psychoeducation • The Cycle of anxiety psychoeducation • Personal Stories • Summing up	• Understanding My Situation • Staying in the Present • Steps towards your goals (goal-setting)	Common to Space from Depression, Space from Anxiety, Space from Depression and Anxiety
3. Noticing feelings	• Getting started with feelings (video) • Emotions and Your Body quiz • Understanding Emotion psychoeducation • Understanding Physical Sensations psychoeducation • Lifestyle psychoeducation • Personal Stories • Summing up	• My CBT Cycles • Changing your physical sensations • Influences on my mood • Progressive Muscle Relaxation • Steps towards your goal	Common to Space from Depression, Space from Anxiety, Space from Depression and Anxiety
4. Boosting behaviour	• Focus on behaviour (video) • Mood and Behaviour quiz • Beating behavioural traps psychoeducation • My activities psychoeducation • Personal Stories • Summing up	• Getting Motivated • Activity Scheduling • Staying in the Present • Steps towards your goal	If Space from Depression or Space from Depression and Anxiety
4. Managing worry	• Introduction (video) • Anxious Thoughts and Worry quiz • Worry and anxiety psychoeducation • Practical versus hypothetical worries psychoeducation • How to manage our worries psychoeducation • Personal Stories • Summing up	• Worry Tree • Staying in the Present • Steps towards your goal	If Space from Anxiety
5. Spotting thoughts	• Focus on thoughts (video) • Me and My Thoughts Quiz • Negative thinking and mood psychoeducation • Thinking traps psychoeducation • Catching thoughts psychoeducation • Personal Stories • Summing up	• Me and My Thoughts Quiz • My CBT Cycles • Staying in the Present (watching thoughts) • Steps towards your goal	Common to all
6. Challenging thoughts	• Talking back (video) • Your Thinking Style Quiz • Hot Thoughts psychoeducation • Challenging your Thoughts psychoeducation • Tackling thinking traps psychoeducation • Coping with difficult situations • Personal Stories • Summing up	• My CBT Cycles • Staying in the Present • Steps towards your goal	Common to all
7. Managing worry (worry postponement)	• Introduction (video) • Anxious Thoughts and Worry Quiz • Worry and anxiety psychoeducation • Practical versus hypothetical worries psychoeducation • How to manage our worries psychoeducation • Personal Stories • Summing up	• Worry Tree • Staying in the Present • Steps towards your goal	If Space from Depression and Anxiety
7. Core beliefs	• Introduction (video) • Core Beliefs Quiz • What are core beliefs? psychoeducation • Healthy core beliefs psychoeducation • Identifying core beliefs psychoeducation • Challenging core beliefs psychoeducation • Balancing core beliefs psychoeducation • Personal Stories • Summing up	• Uncover your core beliefs • Steps towards your goal	If Space from Depression
7. Facing your fears	• Breaking it down (video) • Facing your fears quiz • Avoidance and anxiety psychoeducation • Graded exposure psychoeducation • Personal Stories • Putting it into action psychoeducation • Summing up	• Facing your fears • Steps towards your goal	If Space from Anxiety
8. Bringing it together	• New ideas in action (video) • Finishing up • Warning signs and planning for wellness • Social support • Moving forward • Personal Stories • Summing up	• Staying Well Plan • Goals • Taking Stock • Staying in the Present • Steps towards your goal	Common to all
Optional modules (can be unlocked and added to all modules)	• Challenging Times • Self-esteem and I • Money Worries • Sleep Difficulties • Relaxation • Employment Support • Behavioural Experiments • Communication and Relationships • Managing Anger • Grief and Loss • Facing Fears (if not already a module) • Core Beliefs (if not already a module)		

The intervention course will be split into modules with participants encouraged to take approximately 1–2 weeks to work through each module. The Internet package sends automated reminders to participants who have not logged into the intervention package each week. The platform is always available for users to access.

The intervention will be identical by content and design between the guided versus unguided versions, except for the provision of support and contact from a CBT-trained therapist (Psychological Wellbeing Practitioner) for the guided version. Psychological Wellbeing Practitioners (PWPs) are a UK-specific workforce specifically trained to be competent in supporting structured low-intensity interventions such as guided CBT following a standardized British Psychological Society (BPS)-accredited nationally commissioned APT training course (e.g. PGCert/GradCert Psychological Therapies Practice-Low Intensity Cognitive Behavioural Therapy), involving 45 days of academic work (one day per week) alongside supervised practice within a local NHS Talking Therapies service over 12 months. Such courses are typically completed by psychology graduates, who deliver low-intensity interventions for anxiety and depression in the Talking Therapies services of the NHS in England. These training courses directly evaluate competence in supporting guided CBT, and thus, any successful course graduate and practising PWP would be eligible to deliver this intervention.

In the guided version, the PWP will welcome the participant to the platform upon signup and offer an initial optional call to discuss their specific difficulties and to tailor treatment; thereafter, the PWP will provide contingent feedback and support when users complete the next intervention module on the platform, with PWP and participant collaboratively agreeing on a timescale to complete the next module and for the next review session, typically after 1–2 weeks. This feedback can take the form of 15–30 min of videoconference or telephone calls or asynchronous written feedback within the treatment platform, tailored to the preference of the participant (i.e. a blended model for many students). Templates will be used for written feedback to ensure fidelity and adherence to the treatment model: for participants who receive a videoconference or telephone call, this will summarize the content of the call; if there is no call, it will review what the participant has done in the Internet platform and refocus them on the next module. The feedback will focus on supporting and encouraging participants, addressing their questions, providing guidance to revisit sections or practice particular exercises and giving access to relevant supplementary modules. The PWPs will monitor sign-up to the unguided platform and will send reminders to students who have not accessed the intervention.

### Intervention adherence

The use of the i-CBT platform will be assessed and recorded, including the number of modules that are completed, the usage of tools within the platform and the number of weekly reviews attended, whether asynchronous feedback or remote videoconferencing. A minimum intervention dose for the app will be defined a priori, with “compliance” defined as completion of a pre-specified minimum level of usage of the i-CBT (at least 4 modules completed of the core 8 modules in the treatment package).

### Outcomes

Outcomes will be assessed at baseline (pre-randomization) and 3 months post-randomization.

#### Primary outcome

The primary outcome measures will be the mean symptoms of anxiety (GAD-7) and depression (PHQ-9) at 3 months (primary endpoint) adjusted for baseline scores.

#### Secondary outcomes

Secondary outcomes include the mean scores at 3 months adjusted for baseline scores: the Warwick-Edinburgh Mental Wellbeing Scale (WEMWBS) [47], a widely used and well-validated measure of wellbeing; PSWQ-S [40] and RRS-B [41] to measure worry and rumination; and the Work and Social Adjustment Scale (WSAS) [48], a frequently used measure of functioning with respect to work/education, home management, social leisure, private leisure, and close relationships, each rated from 0 not at all impaired to 8 severely impaired. The Brief Resilience Scale [49] will assess students’ self-reported resilience. A bespoke questionnaire will assess the mean use of university-based and non-university-based healthcare (including National Health Service) services, resources, and support during the 3-month follow-up period. Another bespoke questionnaire will ask participants to report on the actual academic marks and their academic targets for the previous year and whether they were satisfied with their academic progress.

Participants will be asked to complete brief measures each week for 8 weeks after randomization to provide potential mediators of change: a two-item measure of depression (PHQ-2) [50]; a two-item measure of anxiety (GAD-2) [51]; a single-item measure of stress over the last week (scored 0 none to 4 very severe); six items from the Cognitive and Behavioural Response to Stress Scale [52] assessing the frequency and usefulness of use of cognitive reinterpretation, behavioural activation, and relaxation or meditation strategies; 3-item adaptations of the automaticity component of the Self-Report Habit Index [53] focused on assessing the extent to which worry and problem-solving respectively are each a habit (e.g. “something that I do automatically” “I do without thinking”) over the past 7 days; 2-items from the Self-Compassion Scale-short-form [54]; 2 items from the RRS-Brooding 5-item questionnaire [41]; a one-item measure assessing self-efficacy (“In the last seven days I feel better prepared to handle situations I could not handle before” rated using a seven-point Likert scale ranging from − 3 (“absolutely not true), 0 (“neither nor”) to + 3 (“absolutely true”) and a one-item measuring problem clarification (“In the last seven days, I understand myself and my problems better” rated using a seven-point Likert scale ranging from − 3 (“absolutely not true), 0 (“neither nor”) to + 3 (“absolutely true”).

The following descriptive variables will be assessed only at baseline: age, gender, sexuality, year of study, course of study, ethnic group, educational level. The extensive battery of potential prescriptive predictors to inform the development of an ITR will only be assessed at baseline.

### Sample size

The sample size for the efficacy question was calculated using the co-primary outcomes based on a minimum clinically important difference (MCID) at primary endpoint allowing for power at 0.80 and a two-sided alpha at 0.05 and using a Bonferroni correction for the two tests. The co-primary outcomes are symptoms of depression (PHQ-9) and anxiety (GAD-7) at 3 months follow-up. Based on previously established MCID for these measures (PHQ-9, MCID 2.59, SD = 5.4; GAD-7, MCID 4, SD = 5.07) [55, 56] and assuming 20% follow-up attrition, the calculated sample sizes are *n* = 129 per arm and *n* = 41 per arm respectively, with the larger of these (n = 129 per arm) the one required to satisfy the power requirements for both co-primary outcomes after the Bonferroni correction, i.e. a total sample of *n* = 258.

However, to power the study for the development of an ITR, we followed the recent recommendation arising from machine learning stimulations, which suggested that at a power of 0.80 based on a two-sided alpha of 0.05, a sample of at least 300 patients per arm is needed to develop an ITR that can detect a clinically meaningful difference in remission rates (between 5 and 10%) assuming a baseline remission rate from depression of 30% from both treatments aggregated [57]. As such, we increased the sample size to 300 participants per arm as the minimum to obtain stable estimates and generate a reliable ITR. This sample size gives a power of greater than 99% to compare the efficacy of guided versus unguided i-CBT.

### Statistical analysis plan

The primary analyses will be intention-to-treat (ITT) analyses [58] (i.e. all participants will be included in the analyses according to their randomized allocation irrespective of intervention adherence) and based on participants with complete outcome data at 3-month follow-up. Where necessary, we will adjust for baseline outcome scores and stratification variable (site). The primary inferential analyses will compare across trial arms for the primary and continuous secondary outcomes at 3 months follow-up, adjusted for baseline scores, using multilevel mixed-effect models, which enable us to examine nested hierarchies in the data (individual, intervention, university), across time (examining pre-to-post change), to capture dependencies in the data, to investigate individual trajectories (random intercepts, random slopes), and which have less restrictive assumptions re missing data.

For the purposes of precision models, we will first explore which pre-treatment measures moderate the differential effects of treatment condition. Because individual predictors are usually weak and underpowered, our primary approach will be to use composite models that combine individual moderators to produce ITRs that can guide the selection of treatments most likely to be helpful for individual students. The approach will follow precedents established for developing precision treatment models via cross-validated machine learning methods as used in similar studies [30, 59–61]. Such machine learning methods for estimating heterogeneity of treatment effects include extensions of the generalized random forest algorithm [62–64] and of the Super Learner algorithm [65, 66], an ensemble machine learning approach. We will use the generalized random forest algorithm [62–64], with the option to add machine learning approaches (including ensemble approaches) as necessary, to develop a model that best predicts the relative effectiveness of alternative interventions. The machine learning approach has been validated for developing effective composite models, e.g. developing preliminary ITR with *n* = 150 [57, 61] and has the advantages of avoiding the need to specify main effects correctly and allowing the data‐driven estimation of nonlinear and higher‐order complex interactions across predictors. The value of guided and unguided iCBT at the individual level will be quantified using a counterfactual approach estimating the attained improvement of the mean outcome under a treatment selection scheme that always selects the treatment option with the best predicted outcome compared to the mean outcome under balanced randomization, generating a difference score or a personalized advantage index [67].

Secondary analysis will be undertaken including:

- Complier Average Causal Effect (CACE) analysis [68, 69] to provide an estimate of a treatment effect accounting for pre-specified per-protocol adherence and compliance with the treatment, whilst retaining the benefits of randomization.
- The extent of missing data will be considered, and where necessary, in agreement with the Trial Steering Committee (TSC), imputation (e.g. doubly robust estimation combining propensity score models with multiple imputation to reduce risk of bias) will be used to adjust for loss to follow-up.
- Exploratory mediation analyses will be undertaken to gain insight into mechanisms that could explain the potential effect of the interventions on primary outcomes. We will use modern causal inference methods using structural equation modelling or parametric regression models to assess mediation effects [70]. For mediation analyses of longitudinal data, assuming ICC = 0.5, the sample sizes to achieve MCID calculated above can detect small effect sizes for mediators (β = 0.14) in RCTs (with up to 8 micro-repeated assessments), at power = 0.80. A series of models will be performed for each outcome, investigating the mediation effect of each mediator variable individually and the mediation effect of each mediator variable in an overall model combining all mediators. The mediation effect will be reported as the indirect effect of treatment via the specified mediator, with a 95% confidence interval and the proportion of the total intervention effect mediated. Analyses will include the use of instrumental variables to account for effects of unobserved confounding on mediators [68].
- In addition, the ITR analysis will include composite baseline predictors estimated using nested cross-validation machine learning methods, to assign each participant a predicted probability of adherence to self-guided i-CBT and a separate predicted probability of adherence to guided i-CBT. The use of such composite measures as predictors in developing the ITR will make it possible to distinguish heterogeneity in treatment effects due to predicting differential adherence versus differential effectiveness of the two interventions given adherence.
- The results of all models will be compared to primary analysis complete case ITT results.

Analyses will be undertaken by a statistician blinded to group allocation and using Stata v.17 and R 4.5.0.

### Organization, quality assurance and data management

Research data will be automatically collected in a pseudonymized manner through an electronic data capture system licenced/programmed by Exeter Clinical Trials Unit (ExeCTU). In the first instance, all participants will be directed to the electronic data capture system to provide their data. Participants who respond to follow-ups by telephone will have their primary outcome data entered into the system by a site researcher, and this variation in data collection method will be recorded in the EDC. All data will be kept securely and confidentially and only accessed by specified researchers at the University of Exeter. The central data-management team will use de-identified backups for the monitoring of the overall progress and data quality, following a detailed study-specific Data Management Plan, which is available on request. Ultimately, a comprehensive de-identified dataset will be produced that includes all outcome data.

## Trial status

The trial was registered in ISRCTN, number of identification 56784470. Date of registration: 27 October 2022. The study is currently using trial protocol version 4.0 dated 31st May 2024. Recruitment commenced in July 2023 and is ongoing and due to close within academic year 2024–2025. Data collection will end 4 months later.

## Discussion

There has been growing evidence for increasing prevalence of anxiety and depression amongst university students [1–5]. These common mental health problems are associated with lower academic grades and higher rates of drop-out from university as well as predicting negative impact on future careers. Critically, most students who experience anxiety and depression do not receive evidence-based interventions [6–10]. As such, there is an onus to determine scalable, feasible, and efficacious interventions and to appropriately direct students to the appropriate intervention.

Digital CBT provides one potential route to increasing coverage of efficacious interventions and is increasingly being offered as part of the portfolio of mental health services within UK universities, albeit often in an unguided format. It is therefore of great value to know the relative efficacy of guided versus unguided i-CBT for anxiety and depression in university students as well as to distinguish the subsets of students that are as responsive to self-guided as guided i-CBT, responsive only to guided i-CBT, and unresponsive to either and requiring further stepping up to higher intensity psychological therapy. Identifying an ITR is a key step towards precision treatment personalization and to prepare for definitive trials that test whether treatment allocation using ITRs improves outcomes relative to random allocation or usual practice.

The proposed comparison of guided vs unguided digital CBT is the most useful from a service delivery and cost-effectiveness perspective as it is the one with greatest implications for university wellbeing services implementation. Similar i-CBT packages are increasingly becoming a routine offer in UK universities, without knowing if they are being offered to the right students or if supported versions would do better if targeted appropriately. In the context of both increasing mental health demand in universities and constrained finances in higher education institutions, a better understanding of what works and for whom has considerable potential for impact in helping to develop more personalized and cost-effective services.

## Supplementary Information

Supplementary material 1. Battery to assess potential prescriptive predictors (additional to outcome measures which provide measure of severity of symptoms and functioning)

Supplementary material 2. Consent

Supplementary material 3.

## Data Availability

Anonymized datasets arising from this trial will be made available after the primary outcomes are published to researchers and other groups via request to a data committee within the trial consortium and via an open access repository. The results will additionally be updated on https://www.isrctn.com/ISRCTN56784470. We plan to communicate trial results through peer-reviewed open access publications and direct reports to TSC, sponsor, and participants.

## Abbreviations

AE: Adverse event
CACE: Complier average causal effect
DMC: Data Monitoring Ethics Committee
DSM-V: Diagnostic and Statistical Manual of Mental Disorders, Fifth Edition
EDC: Electronic Data Capture System
ExeCTU: University of Exeter Clinical Trials Unit
GAD-7: Generalized Anxiety Disorder-7
ITR: Individualized treatment rule
ITT: Intention-to-treat
MDD: Major depressive disorder
MI: Multiple imputations
NHS: National Health Service
NIHR: National Institute for Health Research
NRES: NHS National Research Ethics Service
PHQ-9: Patient Health Questionnaire
PIS: Participant Information Sheet
PWP: Psychological Wellbeing Practitioner
RCT: Randomized controlled trial
SAE: Serious adverse event
TSC: Trial Steering Committee
