# Supplementary material for: A randomized controlled trial of Internet-delivered guided and unguided cognitive behaviour therapy for treating depression and anxiety in UK university students: study protocol for the Nurture-U Internet CBT trial
Source: Trials. 2025 Sep 26;26:366. doi: 10.1186/s13063-025-09023-1 (PMC12465736; doi:10.1186/s13063-025-09023-1)
Supplement: Supplementary file 1 — Supplementary material 1. Battery to assess potential prescriptive predictors (additional to outcome measures which provide measure of severity of symptoms and functioning) [file 13063_2025_9023_MOESM1_ESM.docx]

Appendix 1. Battery to assess potential prescriptive predictors (additional to outcome measures which provide measure of severity of symptoms and functioning)

| **Measure** | **Details, length, citation** |
| --- | --- |
| **Demographics:**  Age, gender (M/F/neither/both); sexual orientation, race/ethnicity, parental educational attainment, topic of study, disability (yes or no) | Standard questions about background |
| **Mental health history:**  (a) prior diagnosis of mental health condition;  (b) lifetime treatment for mental health condition  (c) hospital admission for mental health condition  (d) Whether first-degree relatives ever been diagnosed mental health condition | (a) 1 to 2 items select from dropdown menú, age of first diagnosis  (b) 1 to 2 items, yes or no; nature of treatment received  (c) 1 item, yes or no  (d) 1 item, dropdown menú select options |
| **Co-morbid mental health difficulties:**  (a) mood disorders, anxiety disorder, eating disorders;  (b) suicidality, ideation, suicide attempts, self-harm  (c) alcohol and substance use  (d) sleep quality | Co-morbid symptoms potential prescriptive predictor of treatment response  (a) Composite International Diagnostic Interview screening scales (CIDI-SC) for DSM anxiety and mood disorders (Kessler RC, Calabrese JR, Farley PA, Gruber MJ, Jewell MA, Katon W, Keck PE, Nierenberg AA, Sampson NA, Shear MK, Shillington AC, Stein MB, Thase ME, Wittchen HU. Composite International Diagnostic Interview screening scales for DSM-IV anxiety and mood disorders. Psychol Med. 2013 Aug;43(8):1625-37. doi: 10.1017/S0033291712002334. Epub 2012 Oct 18. PMID: 23075829.)  (i) Lifetime and current major depression. 4 to 22 items, contingent on responses.  (ii) Lifetime and current generalised anxiety disorder. 4 to 19 items, contingent on responses  (iii) Panic attacks. 1 to 9 items  (iv) Mania / bipolar disorder. 1 to 21 items  (v) Anger /irritability. 1 to 3  (vi) Post-traumatic stress disorder. 1 to 8 items  (vii) Social anxiety. 1 to 5  (viii) Eating disorder. Binging 1 to 3 items; Vomiting/purging 1 to 3 items  (b) Columbia Suicide Severity Rating Scale (C-SSRS) adapted. 4 to 5 items. Posner, K., Brown, G. K., Stanley, B., Brent, D. A., Yershova, K. V., Oquendo, M. A., Currier, G. W., Melvin, G. A., Greenhill, L., Shen, S., & Mann, J. J. (2011). The Columbia–Suicide Severity Rating Scale: Initial Validity and Internal Consistency Findings From Three Multisite Studies With Adolescents and Adults. American Journal of Psychiatry, 168(12), 1266–1277. <https://doi.org/10.1176/appi.ajp.2011.10111704>  (c) Alcohol Use Disorders Identification Test for Consumption (AUDIT-C). 3 items. Bush, K., Kivlahan, D. R., McDonell, M. B., Fihn, S. D., & Bradley, K. A. for the Ambulatory Care Quality Improvement Project (ACQUIP). (1998). The AUDIT alcohol consumption questions (AUDIT-C): an effective brief screening test for problem drinking. Archives of Internal Medicine, 158(16), 1789–1795. <https://doi.org/10.1001/archinte.158.16.1789>  Substance use questionnaire adapted from Adapted from Cuijpers P., Auerbach R. P., Benjet C., Bruffaerts R., Ebert D., Karyotaki E., & Kessler R.C. (2019). The world health organization world mental health international college student initiative: an overview. International Journal of Methods in Psychiatric Research, 28(2), Article e1761. <https://doi.org/10.1002/mpr.1761>. 7 items.  (d) Sleep Condition Indicator. 8 item. Espie, C. A., Kyle, S. D., Hames, P., Gardani, M., Fleming, L., & Cape, J. (2014). The Sleep Condition Indicator: a clinical screening tool to evaluate insomnia disorder: BMJ Open, 4(3), e004183. |
| **Physical health and lifestyle questions**  (a) Height  (b) Weight  (c) Rating of overall physical health (very poor to very good)  (d) Interference of physical health with functioning (none to very severe)  (e) Frequency of exercise in past month (never to 4+ times a week)  (f) Frequency of smoking in past month (never to 21+ times a day)  (g) Consumption of caffeinated beverage on typical day (never to 4+ times a day)  (h) Frequency of recreation/hobbies (never to 4+ times a week) | Physical health and lifestyle may be prescriptive predictors of treatment response  (a) and (b) used to calculate Body Mass Index.  (a) to (h) each a single item. |
| **Difficult and adverse childhood experiences:**  (a) Childhood loss – bereavement, parental divorce, each yes/no and age occurred  (b) Childhood adversity including emotional, physical, sexual abuse | Childhood loss and adversity is a potential prescriptive predictor of treatment response.  (a) 2 to 4 items (questions about age of loss only asked if yes to experiencing divorce or bereavement as child).  (b) adapted Childhood Experience of Care and Abuse scale. 4 items. Bifulco, A., Brown, G. W., & Harris, T. O. (1994). Childhood Experience of Care and Abuse (CECA): A Retrospective Interview Measure. Journal of Child Psychology and Psychiatry, 35(8), 1419–1435. |
| **Current stress:**   1. Perceived stress 2. Current stress including academic stress, social stress, financial concerns 3. Adverse experiences in past 3 months, ratings of frequency and stress | Current stress is a potential prescriptive predictor of treatment response.  (a) Perceived Stress Scale. 4 items. Cohen S, Kamarck T, Mermelstein R. Perceived stress scale. Measuring stress: A guide for health and social scientists. 1994;10(2):1-2.  (b) Post-Secondary Student Stressors Index (PSSI) – Modified. 10 items Linden, B. & Stuart, H. (2019). Psychometric assessment of the Post-Secondary Student Stressors Index (PSSI). BMC Public Health, 19, 1139. <https://doi.org/10.1186/s12889-019-7472-z>  (c) modified from Adverse Events Questionnaire: 2 items. Carver, C. S. (1998). Generalization, adverse events, and development of depressive symptoms. Journal of Personality, 66(4), 607–619. [https://doi.org/10.1111/1467-6494.00026](https://psycnet.apa.org/doi/10.1111/1467-6494.00026) |
| **Coping skills and Self-belief:**  (a) Resilience  (b) Self-esteem  (c) Hopelessness  (d) Self-control | Different coping skills may be prescriptive predictors of treatment response. Includes Rumination and worry assessed in screening.  (a) Brief Resilience Scale. 6 items. Smith, B. W., Dalen, J., Wiggins, K., Tooley, E., Christopher, P., & Bernard, J. (2008). The brief resilience scale: Assessing the ability to bounce back. International Journal of Behavioral Medicine, 15(3), 194–200.  (b) Rosenberg self-esteem measure, shortened to 2 items. Tambs, K., & Røysamb, E. (2014). Selection of questions to short-form versions of original psychometric instruments in MoBa. *Norsk Epidemiologi*, *24*(1-2). <https://doi.org/10.5324/nje.v24i1-2.1822>  (c) Brief-Neg-H measure. 2 items. Fraser L, Burnell M, Salter LC*, et al*  Identifying hopelessness in population research: a validation study of two brief measures of hopelessness BMJ Open 2014;**4:**e005093.  doi: 10.1136/bmjopen-2014-005093  (d) Brief Self-Control Scale. 5 items. Tangney JP, Baumeister RF, Boone AL. High self-control predicts good adjustment, less pathology, better grades, and interpersonal success. J Pers. 2004 Apr;72(2):271-324. doi: 10.1111/j.0022-3506.2004.00263.x. PMID: 15016066. |
| **Loneliness and Social support:**  (a) Loneliness  (b) Social support and social competence | (a) UCLA Loneliness Scale. 4-item. Roberts, R. E., Lewinsohn, P. M., & Seeley, J. R. (1993). A Brief Measure of Loneliness Suitable for Use with Adolescents. Psychological Reports, 72(3_suppl), 1379–1391. <https://doi.org/10.2466/pr0.1993.72.3c.1379>  (b) REsilience scale for ADolescents (READ) - Social Resources and Social Competence Subscales. 10 items. von Soest, T., Mossige, S., Stefansen, K., & Hjemdal, O. (2009). A Validation Study of the Resilience Scale for Adolescents (READ). Journal of Psychopathology and Behavioral Assessment, 32(2), 215–225. https://doi.org/10.1007/s10862-009-9149-x |
| **Personality and Attachment style:**  (a) Emotional reactivity  (b) Personality traits: neuroticism, agreeableness, conscientiousnes, extraversión, openness  (c) Alexithymia  (d) Interpersonal attachment style: secure, dismissive, preoccupied, fearful | (a) Emotional Reactivity Scale selected 2 items highest loading. Nock MK, Wedig MM, Holmberg EB, Hooley JM. The emotion reactivity scale: development, evaluation, and relation to self-injurious thoughts and behaviors. Behav Ther. 2008 Jun;39(2):107-16. doi: 10.1016/j.beth.2007.05.005. Epub 2007 Oct 29. PMID: 18502244.  (b) Big Five Inventory-10 (BFI-10). 10 items. Rammstedt & John (2007) Journal of Research in Personality  (c) Toronto Alexithymia scale. 3 items selected. Bagby, R. M., Parker, J. D., & Taylor, G. J. (1994). The twenty-item Toronto Alexithymia Scale–I. Item selection and cross-validation of the factor structure. Journal of Psychosomatic Research, 38(1), 23–32. <https://doi.org/10.1016/0022-3999(94)90005-1>  (d) Relationship Questionnaire. 4 items. Bartholomew, K. & Horowitz, L. M. (1991). Attachment styles among young adults: A test of a four- category model. Journal of Personality and Social Psychology, 61, 226-244. |
| **Cognitive Abilities and Functioning:**  (a) Self-reported concentration and memory | (a) PROMISs Applied Cognitive-Abilities scale. 4 items. Saffer et al., (2015). Assessing cognitive impairment using PROMIS applied cognition-abilities scales in a medical outpatient scale. Psychiatry Research 226, 169-172. |
| **Orientation and approach towards intervention:**  (a) Motivation to change  (b) Therapy expectancy and preference  (c) Attitudes towards online interventions.  (d) Therapy expectation | (a) Participant specifies a goal for therapy and then rates for effort, importance, confidence to change. 4 items. Miller WR, Johnson WR. A natural language screening measure for motivation to change. Addict Behav. 2008 Sep;33(9):1177-82. doi: 10.1016/j.addbeh.2008.04.018. Epub 2008 May 9. PMID: 18558466.  (b) Variants of Credibility and Expectancy Questionnaire, each completed with reference to guided versus unguided i-CBT. 9 items each.  Devilly GJ, Borkovec TD. Psychometric properties of the credibility/expectancy questionnaire. J Behav Ther Exp Psychiatry. 2000 Jun;31(2):73-86. doi: 10.1016/s0005-7916(00)00012-4. PMID: 11132119.  (c) Attitudes towards Psychological Online Interventions scale edited. 10 items. Schröder J, Sautier L, Kriston L, Berger T, Meyer B, Späth C, Köther U, Nestoriuc Y, Klein JP, Moritz S. Development of a questionnaire measuring Attitudes towards Psychological Online Interventions-the APOI. J Affect Disord. 2015 Nov 15;187:136-41. doi: 10.1016/j.jad.2015.08.044. Epub 2015 Aug 28. PMID: 26331687.  (d) Single open text item in which participant writes “What do you expect from participating in this study and using this online therapy?” |
| **Problem description** | Participant asked in open text box to “Describe a current problem that you are facing – please use at least 3 sentences to describe the nature of the difficult”. Responses to be rated for positivity-negativity; self-focus; abstract vs concreteness |
