# Supplementary material for: A randomized controlled trial of Internet-delivered guided and unguided cognitive behaviour therapy for treating depression and anxiety in UK university students: study protocol for the Nurture-U Internet CBT trial
Source: Trials. 2025 Sep 26;26:366. doi: 10.1186/s13063-025-09023-1 (PMC12465736; doi:10.1186/s13063-025-09023-1)
Supplement: Supplementary file 2 — Supplementary material 2. Consent [file 13063_2025_9023_MOESM2_ESM.docx]

**Nurture-U Studies of interventions to improve wellbeing, confidence, resilience and mental health in UK University students: Reducing Worry, Internet CBT & Bounce Back**

**This is a summary of the currently active Nurture-U trial called Internet CBT (two other trials have now closed to recruitment).**

**More information can be found in the Participant Information Sheet on page 2.**

**What is this remaining trial investigating?**

The Nurture-U trial is conducted online with no ‘in-person’ requirement. The trial is called Internet CBT and is comparing therapist-supported versus self-directed internet cognitive behavioural therapy for anxiety and depression to help university students.

**Who can take part?**

Students with elevated levels of anxiety and/or depression, who are over 16 years old and from any UK university. Some students may not be suitable for this trial but our system will let you know this.

*See Page 1*


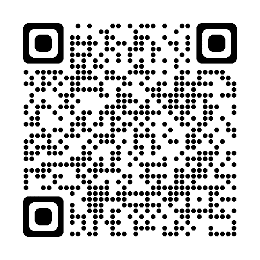


**Where can I get more information?**

Visit [Research Trials | Nurture-U (nurtureuniversity.co.uk)](https://www.nurtureuniversity.co.uk/research-trials) to read more about each trial,

or scan our QR code

Page **2** has our contact details.

**What would taking part involve?**

- Complete a brief online consent form (approx. 5 mins)
- Answer ‘screening’ questions online about demographics, symptoms, and coping styles (approx. 5 -10 mins)
- Our system checks if you are eligible for the trial
- Complete a second online consent form agreeing to participate in the trial (approx. 5 mins)
- Answer ‘baseline’ questions online (approx. 45 - 60 mins)
- Our system automatically randomises you to one of two groups in your trial
- Answer questions online each week for 8 weeks (1-2 mins) and at 3 months (approx. 10 mins)
- If you’re ineligible (not suitable) for one of the trials our system will tell you and provide details of helpful resources to access

**Will I benefit?**

We hope the intervention will help you. You will also help other students and could receive £10 electronic shopping vouchers for completing each assessment for us.


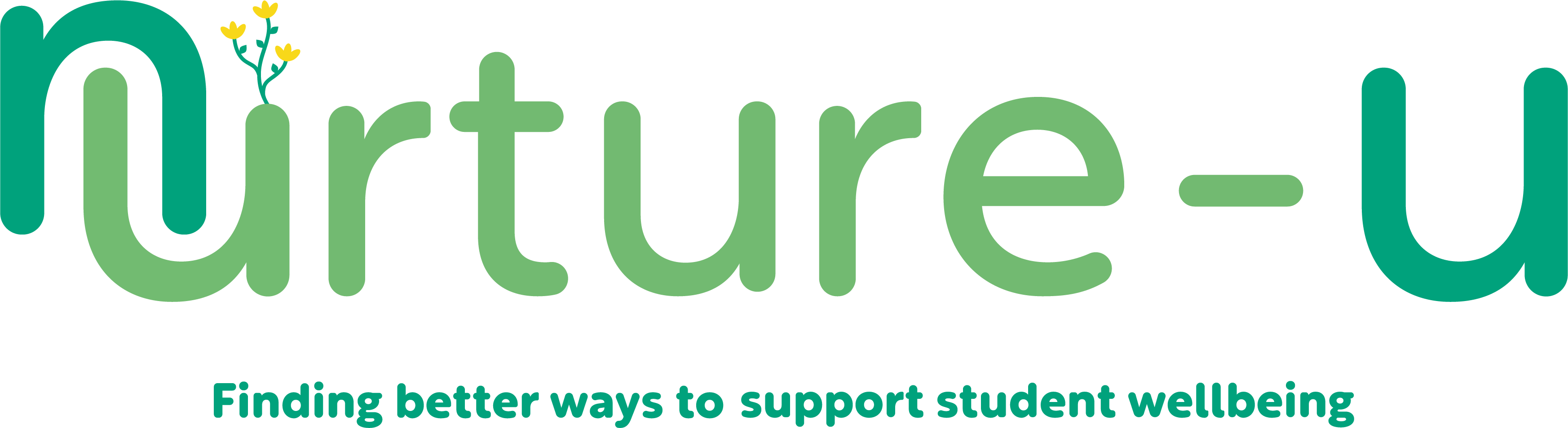


**Page 1**

**Participant Information Sheet: Screening**

**Nurture-U Studies of interventions to improve wellbeing, confidence, resilience and mental health in UK University students**

The Nurture-U project includes several studies to investigate different interventions to help university students. Currently, only a study comparing therapist-supported versus self-directed digital cognitive-behavioural therapy for anxiety and depression remains open to students. We have a brief online survey to assess if you are eligible and to then direct you to the Internet CBT study or to information about why the study is not suitable for you.

**What will I have to do?**

We ask you to complete brief online questions about demographics, symptoms and coping styles. These questions should take less than 5 minutes to complete.

**Who can take part?**

University students (undergraduate or postgraduate above the age of 16) in the UK, principally students at the Universities of Exeter, Oxford, Cardiff, Newcastle, Southampton, or King’s College London (as the participating universities in Nurture-U project), although students at other universities can participate. For the digital cognitive-behavioural therapy study, you need to have elevated levels of anxiety and/or depression.

**Do I have to take part?**

No, it is up to you to decide whether or not to take part. If you agree to take part you will need to sign a consent form online. Taking part is entirely voluntary. If you take part you are free to withdraw at any time without giving a reason.

**Is my data confidential and private?**

All information collected about you will be kept confidential and stored securely and password protected. Your contact details (name, email, telephone number) will be kept separately from all other data. Your responses on the website and the treatment platform will be only linked to a project identification number, not with your contact details, to keep your personal responses private.

**Who will have access to my data?**

Only selected researchers within the Exeter Nurture-U consortium team can access your contact details. This is so they can respond to your questions or requests for help.

After the study ends it may be that academic journals and other research teams will ask for the data from the study to be made public (so they can carry out further research or check our findings). The data they use will not include your name or any of your contact details.

**How long will my data be held?**

**Page 2**

Your data will be held no longer than required (at least 10 years).

**What if I want to withdraw from the study or delete my data?**

You can withdraw from completing the screening assessment at any time without giving us a reason, but we will keep information about you that we already have. Researchers need to manage your data in specific ways for the research to be reliable. This means that they won’t be able to let you see or change the data they hold about you. Research could go wrong if data is removed or changed.

**What help is provided?**

If you report increased distress or thoughts of harming yourself on our assessment website, we will give you automated advice and point you to sources of help. You can also contact us for further advice at any time and we will (wherever possible) respond within 2 working days.

**What will happen to the results?**

We will analyse the information received from all those taking part together at the end of the study. We aim to publish the work in academic journals. We will write our reports in a way that no-one can work out that you took part in the study. We will publish a summary of the findings on our website.

**Who has reviewed the study?**

This study has been reviewed and given a favourable opinion by the Psychology Research Ethics Committee at the University of Exeter and the contacts for this committee are Co-Chairs, Ciro Civile (c.civile@exeter.ac.uk) and Ian McLaren (I.P.L.McLaren@exeter.ac.uk).

**What if I have more questions?**

If you have any questions about taking part in the trial, please contact the Nurture-U Trial Manager, Dawn Phillips: telephone 07890 384247 and email Nurture-U@exeter.ac.uk

*Finding better ways to support students*

**Consent Form A: Screening**

Nurture-U Studies of interventions to improve wellbeing, confidence, resilience and mental health in UK University students

**Please tick box**

1) I understand that I am consenting to complete online questionnaires and tasks to check if taking part in a Nurture-U intervention study is right for me.

2) I understand that all my data will be kept confidential and stored securely. My contact

details will be kept separately from other information. My data will be held no longer than required (at least 10 years).

3) I understand that anonymised and de-identified data collected from me (when there is no longer any link with any identifying details such as my name, contact details) will be shared with researchers and collaborators within the Nurture-U project and may be used to support other research in the future.

4) I understand that my participation is voluntary. I am free to withdraw at any time without giving any reason, but the study team will keep information about me that they already have.

5) I am aged 16 or over and am a university student.

6) I confirm that I have read the screening information sheet dated 12.11.2024 Version 3.0 and I have been provided with a phone number and email address so that I can ask any questions.

7) I agree to take part in the assessment for the Nurture-U intervention study. I understand that it is possible that I will be informed that taking part is not right for me.

Name of Participant Date Signature

Autocomplete

Name of Person

taking consent Date Signature

Please provide your University Email address: Phone number*:*

*The consent form and info sheet are now automatically sent to the ppt by email after completion and before this point.*

**Internet-based cognitive-behavioural therapy for acute depression and anxiety in UK university students**

**This is a summary of the Nurture-U Internet CBT trial.**

**More information can be found in the Participant Information Sheet (PIS) on page 2**

**Who can take part?**

All UK university students over 16 years old who have elevated anxiety and/or depression & no diagnosis of bipolar disorder or psychosis.

Our initial screening process will check if you are eligible (suitable). If you are not eligible you will be informed.

*See Page 3*

**Can I withdraw from the trial?**

Taking part is voluntary. You can withdraw at any time without giving a reason. We will retain any data we already have for the research to be reliable.

*See page 5*

**What would taking part involve?**

- Answer ‘baseline’ questions online about symptoms, background, health history, life experiences, stress, personality (approx. 45 - 60 mins)
- Work through the digital therapy for 6-8 weeks and put it into practice in daily life (approx. 1 hour each week)
- Answer questions online each week for 8 weeks to track your progress (1-2 mins)
- Answer questions online at 3 months to see if the therapy was helpful (approx. 10 mins)
- You have 50% chance of working through the therapy yourself and 50% chance of support from a therapist via telephone, Teams, or written feedback. *See page 2*

**What data will you keep about me?**

You will be given a unique study number to keep your contact details separate from any data you provide in the questionnaires or treatment (i.e., pseudo-anonymised). We need your name, email, mobile number to do the research and keep in touch. This personal data will be stored for no longer than required (at least 10 years) and will be kept strictly confidential, private, and secure, unless you report risk to yourself or others, where we may contact your doctor or relevant services. Anonymised data will be stored indefinitely. *See page 4 and our Privacy Personal Data Protection Policy.*

Page 5 has our contact details.

Visit <https://www.nurtureuniversity.co.uk/anxietyanddepression> to read more about this trial

This trial has been reviewed and approved by the Psychology Research Ethics Committee at the University of Exeter.

**Are there any risks?**

There are no known risks - you can contact the research team if there are any problems.

*See Page 5*

**Will I benefit?**

We hope the therapy will help you. You will also help other students & receive £10 electronic shopping vouchers each for completing all 8 weekly measures and the 3-month follow-up.

*See Page 3*

**Why is the research being done?**

Digital CBT is an effective treatment for depression and used widely in universities. We want to find out which students do well with it on their own or which only benefit with support from a therapist. The study will help us know what works for whom and better plan student mental health services.

*See Page 2*


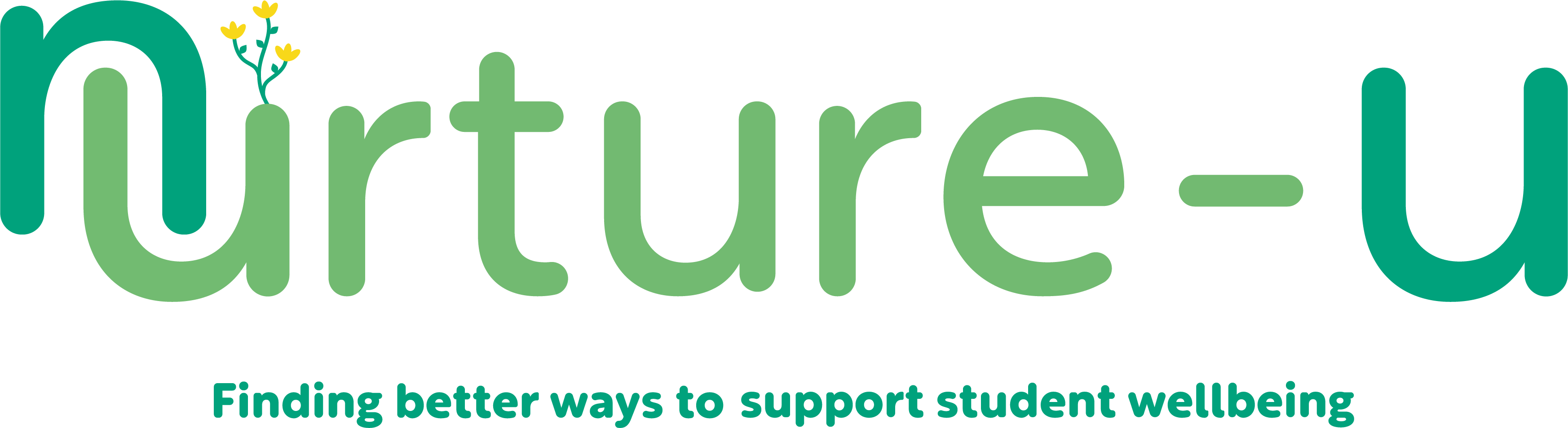


**Page 1**

**Internet CBT Participant Information Sheet**

**TITLE: Internet-based cognitive-behavioural therapy for acute depression and anxiety in UK university students**

**What is this study about?**

This project seeks to find out which forms of digital therapy students prefer and to understand which therapy works best for whom. Many universities offer some form of digital cognitive-behavioural therapy (CBT) for students who have anxiety and/or depression. This is because there is good evidence that these cognitive-behavioural therapy treatments can be effective. These treatments usually involve a combination of changing what you do (e.g., building up positive activities, reducing avoidance of anxiety-provoking situations) and changing how you think (e.g., spotting and challenging negative thoughts). These therapies can be offered as a treatment that the individual works through on their own or with support from a mental health professional. Some people prefer to complete the treatment on their own: there are no waiting lists, it is completely private, and you can access it when you want. However, for other people, support from a professional may be necessary and a critical part of helping them to recover.

​

We want to find out who does well with digital CBT on their own and who would only benefit when they have support from a therapist. Digital CBT without a therapist can be offered to a really large number of students as there is no limit on capacity based on therapist availability or scheduling, so it is important to know who can benefit just as well from this approach. Equally, we want to know who really needs the support of a therapist so we can make sure they get the right help. This research will therefore help us to improve services by better directing students to the right treatment, and by better planning and resourcing the services required to help the maximum number of students.

​

Taking part will thus help us improve treatment services for other students.

**What will I have to do?**

We ask you to complete brief questions about symptoms of anxiety and depression and well-being twice online. These questions will be asked at the start of the study and then at a follow-up after 3 months. These measures should take about 10-15 minutes to complete each time. We also ask you to complete very brief measures (taking about 1-2 minutes) once a week for the first 8 weeks after the baseline. These measures will help us to understand what is helpful or not helpful.

At the start of the study, we will ask you to complete a comprehensive set of questions about your physical and mental health, prior life experiences, demographics, family background, current and past stress, personality and coping skills, social relationships, and attitudes to and experience of therapy. This will take about 30-40 minutes to complete. These questions are critically important because they will help us to predict who might do better in which version of the digital CBT therapy. These questions have been found to predict who does well in therapy before and so we are using them to develop statistical models of who might benefit from the different versions of CBT.

**Page 2**

We will offer half of the study participants the option of using the digital cognitive-behavioural therapy to reduce anxiety and depression on their own (self-directed) and half of the study participants the option to use digital cognitive-behavioural therapy with the support of a therapist. This support could take the form of video-conferencing, emails or online conversations within the digital therapy platform, approximately once a week. The therapy typically takes 6-8 weeks to complete and we recommend completing one module a week plus practice in between each module. This allocation will be decided by chance (at random). This is so we can learn who might benefit most from each version of the therapy.

**What payment will I receive?**

As a thank you and reward for completing each follow-up assessment you will be sent electronic shopping vouchers worth £10 by email for completing all of the 8 weekly measures and after you complete your 3 month follow ups (i.e., up to a maximum of £20).

**Why take part and what are the possible advantages of taking part?**

By taking part, you will play a major role in improving well-being and mental health for university students. Taking part may help you learn about, understand, and better manage your own anxiety and depression. The digital treatments have been proven to be on average effective and so they may be of benefit to you. These interventions are provided to you for free. It will also help us to improve treatment and services for anxiety and depression for other university students.

**Who can take part?**

University students (undergraduate or postgraduate, above the age of 16) in the UK, principally students at the Universities of Exeter, Oxford, Cardiff, Newcastle, Southampton, or King’s College London (as the participating universities in Nurture-U project), although students at other universities can participate. We will ask you to complete online questionnaires to see if the study is likely to be right for you. To be eligible you need to be reporting elevated levels of anxiety and/or depression symptoms during our initial screening questions. If you have ever had a diagnosis of bipolar disorder (mania) or psychosis, you cannot take part because the intervention is not designed for these conditions. If you can’t take part, our automated pages can direct you to other sources of help and advice, and you can find more information at our website [www.nurtureuniversity.co.uk](http://www.nurtureuniversity.co.uk).

**Allocation to the digital CBT therapy**

All eligible individuals within the study are selected at chance to either receive the digital CBT therapy without support (1/2 chance) or to receive the digital CBT therapy with support (1/2 chance). This treatment will be accessed through an internet platform. It involves a number of modules that you can work through to learn new strategies and try out new skills. By comparing these conditions, we will find out if we can predict who benefits most from each treatment over 3 months.

**How do I access the digital treatment?**

If the study is right for you and you consent to take part, one of our team will set you up on the digital platform and inform you whether you are receiving therapist support or not. You will receive a link via email to sign in and set up your own password. You will need to have access to the internet via mobile data or Wi-Fi for the treatment to work.

**How long would I need to use the treatment for?**

**Page 3**

You can use the treatment for as often as you like during the 3 months in the trial. We recommend working through all the treatment modules to get the most benefit. Most people find that this works best if you do one module a week and then practice the skills in between.

**Do I have to take part?**

No, it is up to you to decide whether or not to take part. If you agree to take part you will need to sign a consent form online. Taking part is entirely voluntary. If you take part, you are free to withdraw at any time without giving a reason.

**How will we use information about you?**

We will need to use information from you for this research project. This information will include your name, contact details (email, mobile number). We will use this information to do the research or to check your records to make sure that the research is being done properly.

We will keep all information about you safe and secure. Once we have finished the study, we will keep some of the data so we can check the results.

**Is my data confidential and private?**

All information collected about you will normally be kept strictly confidential. The main exception is if in an email or a telephone call with us, you reported significant difficulties or risk of harm to yourself. In this case, we may contact your doctor (if you provide their contact details).

Your information will be kept confidential and stored securely and password protected. Your contact details (name, email, telephone number) will be kept separately from all other data that you provide to us such as your responses on the website and the treatment platform. This data will be only linked to a project code number, not with your contact details, to keep your personal responses private and confidential.

**Who will have access to my data?**

Only selected researchers within the Exeter Nurture-U consortium team can access your contact details. This is so they can contact you about follow-ups, to arrange for vouchers to be sent out, or to respond to your questions or requests for help. Other project researchers will only have access to the information you enter into the assessment website and the treatment platform, linked to a code number. People who do not need to know who you are will not be able to see your name or contact details. In these cases, your name and contact details are replaced with a code number in the research data. That means that specific data can only be linked to a unique individual when this data is combined with other pieces of data, which are stored and protected separately. This is called in technical terms ‘pseudonymised data’.

The third-party electronic shopping voucher provider will be given your name and email address only but no other information so they can send you access to the vouchers you have earnt. Your contact details will not be used by any party for sales or marketing purposes.

The data you enter when you complete your screening, baseline assessment and follow-ups will be collected and stored in a secure password protected information technology system managed by the University of Exeter clinical trials unit. Data from the digital therapy will be collected and stored by our subcontractor who provide the therapy platform, before being sent to the Exeter Clinical Trials Unit.

The University of Exeter as the sponsor of the trial is the data controller with respect to your personal data, and as such will determine how your personal data is used in the study. The provider of the digital therapy platform is a data processor with respect to your personal data and processes this data as instructed by our research team. As data controller, the University will take the appropriate measures to ensure your data is collected, transferred, processed, and stored according to relevant data protection legislation, including ensuring appropriate due diligence is performed on our data processors (who may perform tasks on our behalf, such as survey software or provide online therapy platforms).

**Page 4**

Our research team and the digital therapy provider will process your personal data for the purpose of the research outlined above. Research is a task that we perform in the public interest. Further information about your rights with respect to your personal data is available from our privacy and personal data protection policy.

We will collect data on how you use the digital therapy. This includes how often you use it, which elements you use, etc. We will also collect your responses in the digital therapy, such as the scores or text answers you enter. We collect this data so we learn what is helpful or not in the digital therapy and improve it.

The website and the digital therapy routinely collect technical data such as hardware configuration, software configuration, access provider, data exchanges and site usage. We collect this so that we can run the website and digital therapy efficiently and keep your data secure. Technical data will not be linked to your contact information unless you ask us for technical support as we will need to contact you to provide help.

Free-text responses you provide in the digital therapy or online assessments may be used in the form of anonymous quotes on our advertisements, promotional materials, social media accounts or in future publications. You will not be named or identified in any quotes and any references to named individuals or contact details will also be removed.

After the study ends it may be that academic journals and other research teams will ask for the data from the study to be made public (so they can carry out further research or check our findings). The data they use will not include your name or any of your contact details.

**How long will my data be held?**

Your data will be held no longer than required (at least 10 years). To safeguard your rights, we will use the minimally required Personal Identifiable Data possible. Our data protection and privacy policy is provided with this information sheet here.

**What if I want to withdraw from the study or delete my data?**

You can withdraw from using the digital intervention or taking part in the trial at any time without giving us a reason, but we will keep information about you that we already have. Researchers need to manage your data in specific ways for the research to be reliable. This means that they won’t be able to let you change the data they hold about you. Research could go wrong if data is removed or changed.

**What are the possible disadvantages of taking part?**

Taking part involves giving of your time to complete the questionnaires and use the digital therapy. Because some of the questions in the assessment ask about past and present negative emotions and difficult experiences, and the therapy asks you to tackle current difficulties, there is a small chance that this may produce mild and brief upset if you are reminded of an unpleasant event. However, this would be no more than usually experienced in daily life. We are not aware of any other side effects, disadvantages, or risks of using the digital treatment.

**What help is provided?**

If you report increased distress or thoughts of harming yourself on our assessment website, we will give you automated advice and point you to sources of help. If you report increased distress or thoughts of harming yourself in the treatment platform in the supported condition, the therapist will contact you to provide relevant support. You can also contact us for further advice at any time and we will (wherever possible) respond within 2 working days. Your GP (family doctor) remains responsible for your medical care. It is your responsibility to contact them for further help.

**Page 5**

**What will happen to the results?**

We will analyse the information received from all those taking part together at the end of the study. We aim to publish the work in academic journals. We will write our reports in a way that no-one can work out that you took part in the study. We will publish a summary of the findings on our website. We will also send this report to all participants who request it.

**Who has reviewed the study?**

This study has been reviewed and given a favourable opinion by the Psychology Research Ethics Committee at the University of Exeter and the contacts for this committee are Co-Chairs, Ciro Civile ([c.civile@exeter.ac.uk](mailto:c.civile@exeter.ac.uk)) and Ian McLaren ([I.P.L.McLaren@exeter.ac.uk](mailto:I.P.L.McLaren@exeter.ac.uk)).

**What if I have more questions?**

If you have any questions about taking part in the trial, please contact the Nurture-U Trial Manager, Dawn Phillips: telephone 07890 384247 and email [Nurture-U@exeter.ac.uk](mailto:Nurture-U@exeter.ac.uk)

If you wish to complain about any aspect of the research team’s work you can complain to the Study Principal Investigator, Professor Edward Watkins, from the University of Exeter, UK.
